# Perinatal mental health in India: protocol for a validation and cohort study

**DOI:** 10.1093/pubmed/fdab162

**Published:** 2021-10-08

**Authors:** G Fellmeth, M T Kishore, A Verma, G Desai, O Bharti, P Kanwar, S Singh, H Thippeswamy, P S Chandra, J J Kurinczuk, M Nair, F Alderdice

**Affiliations:** 1 National Perinatal Epidemiology Unit, Nuffield Department of Population Health, University of Oxford, Oxford, UK; 2 Department of Clinical Psychology, National Institute of Mental Health and Neuro Sciences, Bangalore, India; 3 Department of Obstetrics and Gynaecology, Dr Rajendra Prasad Government Medical College, Kangra, Himachal Pradesh, India; 4 Department of Psychiatry, National Institute of Mental Health and Neuro Sciences, Bangalore, India; 5 State Institute of Health and Family Welfare, Department of Health and Family Welfare, Government of Himachal Pradesh, India; 6 Department of Psychiatry, Dr Rajendra Prasad Government Medical College, Kangra, Himachal Pradesh, India; 7 School of Nursing and Midwifery, Queen’s University Belfast, Belfast, Northern Ireland

**Keywords:** mental health, pregnancy and childbirth disorders, women’s health

## Abstract

**Background:**

Common mental disorders (CMD) are among the largest contributors to global maternal morbidity and mortality. Although research on perinatal mental health in India has grown in recent years, important evidence gaps remain, especially regarding CMD. Our study aims to improve understanding of CMD among perinatal and non-perinatal women of reproductive age across two settings in India: Bangalore (Karnataka) and Tanda (Himachal Pradesh).

**Methods:**

The study is embedded within the Maternal and Perinatal Health Research Collaboration India (MaatHRI). This mixed-methods observational study comprises three consecutive phases: (i) focus group discussions and individual interviews to explore women’s knowledge and seek feedback on CMD screening tools; (ii) validation of CMD screening tools; and (iii) prospective cohort study to identify CMD incidence, prevalence and risk factors among perinatal and non-perinatal women. Results of the three phases will be analyzed using inductive thematic analysis, psychometric analysis and multivariable regression analysis, respectively.

**Conclusion:**

Improving understanding, detection and management of CMD among women is key to improving women’s health and promoting gender equality. This study will provide evidence of CMD screening tools for perinatal and non-perinatal women in two diverse Indian settings, produce data on CMD prevalence, incidence and risk factors and enhance understanding of the specific contribution of the perinatal state to CMD.

## Introduction

Globally, common mental disorders (CMD) including depressive and anxiety disorders are one of the largest contributors to maternal morbidity and mortality.^1^ A disproportionate burden of perinatal CMD is borne by low- and middle-income countries (LMIC).^2^ In India, persisting stigma towards mental disorders, socio-economic deprivation, intimate partner violence, traditional confinement practices after childbirth and women’s low status in society act as risk factors for the development of mental disorders for many women.^3^ The prevalence of perinatal depression in India has been estimated at 22%, though estimates are subject to significant heterogeneity based on the tools used, sample size, study population and location.^4^^,^^5^ Fewer studies have focused on anxiety. One recent study estimated a prevalence of generalized anxiety disorder of 23% among perinatal women in a tertiary care hospital in South India.^6^ Additionally, an estimated 7.6% of women in India experience suicidal ideation in pregnancy—an important contributor to maternal mortality across low-, middle-and high-income settings alike.^7^

Although research on perinatal mental health in India has grown in recent years, important evidence gaps remain.^8^ Firstly, few studies have assessed the psychometric validity of screening tools for perinatal CMD in India.^4^^,^^9^ Screening tools, which have been translated from other languages and cultural settings but have not been validated locally, may be unreliable when used in Indian contexts.^10^ For instance, optimal cut-offs may vary across settings and especially when used in populations living with chronic stress or in conditions of poverty.^11^ Validation studies should examine whether screening tools perform differently in the antenatal and postnatal periods, their reliability among low literacy populations, and any differences according to whether tools are self-completed or questions are read out by interviewers. Secondly, there is a need to broaden the focus of current evidence. For example, post-traumatic stress disorder (PTSD) and anxiety disorders including panic disorder and phobias in the perinatal period have been relatively under-researched in India as elsewhere globally. Similarly, somatization disorder—a common expression of emotional distress among women in India—has not been sufficiently studied among pregnant or postpartum women and represents a further gap. Certain groups—notably rural and marginalized populations, those living in low-income settings and those with low levels of literacy—are among the most vulnerable to experiencing CMD. Yet the voices of these women are seldom heard: these groups remain under-represented and efforts must be made to render future research more inclusive.^5^ Thirdly, systematic studies to assess the prevalence of CMD antenatally are needed: many postnatal mental disorders have their onset during pregnancy, and few studies assess the problem in a prospective manner from early pregnancy through to the postpartum period. Much of the burden could be averted through effective detection and management of CMD antenatally. Finally, there is uncertainty regarding the extent to which perinatal CMD is associated with the perinatal state *per se*, as few studies have directly compared perinatal and non-perinatal women of reproductive age from the same setting.

Addressing these evidence gaps is essential to improve understanding of perinatal CMD in India, facilitate diagnosis and be better positioned to offer appropriate support and treatment to women. Screening for perinatal mental disorders should occur in maternal health and obstetric settings by healthcare workers including midwives, nurses and primary care doctors. In order for screening initiatives to be scalable, tools should therefore be reliable, simple and quick to administer and have established cut-offs to facilitate stepped-care treatment. Managing perinatal CMD can also improve child outcomes, particularly in LMICs where associations between maternal mental disorders and adverse infant outcomes are exacerbated by exposure to additional socio-economic adversities.^12^ The need to identify effective means of detection and management of CMD among women in India has become increasingly urgent in light of the COVID-19 pandemic, which has disproportionately impacted upon women and heightened levels of anxiety and other mental disorders.^13^

## Aims and objectives

This protocol outlines our study on perinatal mental health in two sites in India. Our study aims to improve understanding of CMD among perinatal and non-perinatal women in India, with the long-term goals of developing reliable detection methods to enable early identification, referral and support for women with CMD. The research questions and objectives the study seeks to address are listed in Table 1. The study will assess the following types of CMD: depressive disorders, anxiety disorders, PTSD, somatization disorder and suicidality. The study objectives will be achieved using a mixed-methods observational design comprising three consecutive phases: (i) qualitative study, (ii) validation study and (iii) prospective cohort study, as described in further detail below.

**Table 1:** Study phases with research questions and objectives to be addressed

*Research questions*	*Objectives*
**PHASE 1. Qualitative study (individual interviews and focus group discussions)**	
• Which are the most appropriate and culturally acceptable screening tools for CMD among perinatal and non-perinatal women in the two study settings?	• Identify appropriate and culturally acceptable screening tools for the detection of CMD in two low-income settings in India • Elicit women’s knowledge and understanding of CMD in these settings
**PHASE 2. Validation study**	
• What is the psychometric validity of selected CMD screening tools among perinatal and non-perinatal women in these settings?	• Validate screening tools for CMD in perinatal and non-perinatal women against a gold standard.
**PHASE 3. Prospective cohort study**	
• What is the prevalence and incidence of CMD among perinatal and non-perinatal women and what is the likely burden of CMD attributable to the perinatal state? • What are the risk factors for CMDs among perinatal and non-perinatal women and do they differ between the two groups?	• Determine prevalence and incidence of CMD among perinatal and non-perinatal women in two low-income settings in India • Identify risk factors associated with CMD among perinatal and non-perinatal women

## Methods

### Setting and participants

This study is embedded within the Maternal and Perinatal Health Research Collaboration India (MaatHRI), a collaboration between the National Perinatal Epidemiology Unit (NPEU), University of Oxford and fifteen hospitals across five states in India.^14^ The study will be conducted by the Departments of Clinical Psychology and Psychiatry, National Institute for Mental Health and Neuro Sciences (NIMHANS), Bangalore, Karnataka; and Dr Rajendra Prasad Government Medical College (DRPGMC), Tanda, Himachal Pradesh. At each site, perinatal women (women who are currently pregnant or up to 12 months post-partum) and non-perinatal women (women who are not currently pregnant and have not given birth within the last 12 months) aged 18-45 years who are willing and able to participate will be invited to participate.

### Phase 1: qualitative study

#### Procedure

The procedures involved in each of the study phases are summarized in Table 2. Phase 1 is a qualitative study consisting of individual interviews and focus group discussions (FGD) to seek feedback on selected CMD screening tools and explore knowledge around CMD. Participants will first complete an individual interview. Women will be shown a selection of CMD screening tools and asked for their views on these tools, including their thoughts on the wording of the questions, the response scales and the perceived user-friendliness of the tool. Next, participants will join an FGD during which the screening tools will be discussed further. Any preferences for certain tools will be explored. We will conduct separate FGD with pregnant, post-partum and non-perinatal women in order to elicit views specific to each of these groups. FGD participants will be recruited from the same settings as participants of the following two phases. CMD screening tools will be selected though discussion between study investigators including psychiatrists and clinical psychologists with extensive experience of conducting mental health interviews with local communities. We will also consult the published literature and consider the results of a systematic review of validated CMD screening tools in perinatal women in India, conducted by members of the study team.^9^ Potential screening tools under consideration are listed in Table 3. Tools, which are not already available in Hindi, Urdu or Kannada, will be translated following WHO guidelines for the translation and adaptation of instruments, including forward-translation and back-translation by a panel of mental health specialists fluent in Hindi/Urdu/Kannada and English.^18^

**Table 2:** Schedule of study procedures

*Procedures*	*Phase*						
	*Phase 1. Qualitative study*	*Phase 2. Validation study*		*Phase 3. Cohort study*			
		*Cognitive interview*	*Validation*	*Visit 1 (EGA <20)*	*Visit 2 (EGA >28)*	*Visit 3 (3m PP)*	*Visit 4 (6m PP)*
Socio-demographic questionnaire	X		X	X			
Focus group discussion	X						
Individual interview	X	X					
Screening tools		X	X	X	X	X	X
Diagnostic interview			X				
Expected duration	6 months	12 months		24 months			

Abbreviations: PP, post-partum; EGA, estimated gestational age (weeks); m, month

**Table 3:** Potential screening tools to be used in study

*Disorder*	*Potential screening tools*
Depression	Patient Health Questionnaire (PHQ-9)^15^ Edinburgh Postnatal Depression Scale (EPDS)^16^
Anxiety	Perinatal Anxiety Screening Scale (PASS)^17^ Generalized Anxiety Disorders scale^18^ (GAD-7)^19^ Anxiety subscale of the EPDS^20^
Suicidality	Suicide Behaviour Questionnaire-Revised^7^
PTSD	PTSD Checklist (PCL)^21^
Somatization disorder	Patient Health Questionnaire-15 (PHQ-15)^22^ Scale for Assessment of Somatic Symptoms (SASS)^23^

#### Sample size

We will include 24–32 women per study site in Phase 1. At each site, we will conduct a minimum of two FGDs with perinatal women and two FGDs with non-perinatal women. Each FGD will include 6–8 women. The number of FGDs was determined by evidence suggesting that more than 80% of all themes to emerge from group discussions are discoverable within two to three focus groups.^24^ If data saturation has not been achieved, a third FGD will be conducted. A maximum of eight participants per group provides optimal data richness, facilitates group moderation, identification of individual voices within the group, seeking of clarifications and exploration of differences in opinions.^25^

#### Analysis

Audio recordings of individual interviews and FGDs will be transcribed verbatim and translated into English. Inductive thematic analysis of the data will be conducted using the six consecutive phases described by Braun and Clarke (2006): familiarization with the data, generation of initial codes, search for themes, review of themes, definition and naming of themes and production of the report.^26^ A ‘buddy system’ will be used whereby one researcher will analyze and code data first. A ‘buddy’ will use the codes identified by the first researcher and apply these to a sample of the transcripts to check the reliability of coding and enhance consistency. The ‘one sheet of paper’ (OSOP) method of analysis will be used to help with the grouping of issues arising from the data into broader themes.^27^ Analyses will be conducted using *NVIVO* qualitative data analysis software.^28^ Results of this qualitative phase will be used to inform the selection of tools for Phase 2 and the collection of data on potential risk factors and confounders for Phase 3.

### Phase 2: validation study

Phase 2 is a cross-sectional validation study. We will use Phase 1 outcomes to select CMD screening tools to be formally validated against a gold standard. In accordance with WHO guidelines for the translation and adaptation of instruments, we will conduct pre-testing and cognitive interviewing of the tools.^18^ Cognitive interviews will be conducted with a small group of participants who will be asked to complete the screening tools. An interviewer will then go through each item of the screening tools with the participant to explore how they interpreted each question. This will help us to understand whether translated screening tools have been clearly and appropriately translated and assess whether semantic equivalence has been maintained. Any necessary modifications will be made to ensure the tools are culturally appropriate.^18^ Next, we will conduct the main validation component. This will involve a larger group of participants. Each participant will be asked to complete a socio-demographic questionnaire, a number of CMD screening tools and finally a diagnostic interview administered by a trained clinician. The CMD screening tools will be selected on the basis of Phase 1 results. The Mini-International Neuropsychiatric Interview (MINI),^29^ which has been widely used in India and will allow for direct comparison with the National Mental Health Survey of India,^30^ will be used as the diagnostic interview. The MINI will be conducted by an interviewer who is blinded to screening tool results.

#### Sample size

At each site, ten women will be included in cognitive interviews and a minimum of 150 pregnant, 150 postpartum and 150 non-perinatal women will be included in the main validation component. These are the minimum sample sizes recommended to reliably determine psychometric properties and assess the validity and reliability of screening tools.^31^^,^^32^

#### Analysis

Results of cognitive interviews will be in the form of interviewer notes. These will be discussed with expert colleagues and feedback will be used to develop final, translated versions of the screening tools. In the main validation component, women diagnosed with CMD on diagnostic interview will be considered a ‘true’ case. Mean scores on screening tools will be compared between women with and without true CMD to test the construct validity of screening tools.^33^ Sensitivity, specificity, positive and negative likelihood ratios and the proportion of true cases correctly identified will be calculated for each cut-off value of the screening tools. Accuracy of screening tools will be evaluated using a receiver operating characteristic (ROC) curve, and reliability will be assessed using Cronbach’s alpha. Youden’s index, the value at which [(sensitivity + specificity)—1] is maximized, will be used to identify the point of optimal balance between sensitivity and specificity and thus be recommended as a cut-off for use in the local population. Data will be analyzed separately for pregnant, post-partum and non-perinatal women. Statistical analyses will be conducted using *STATA*.^34^

### Phase 3: cohort study

Phase 3 will be a prospective cohort study. At each study site, we will recruit (i) perinatal women (‘exposed’ group) and (ii) non-perinatal women (‘unexposed’ group). Women in the perinatal group will be asked to attend in early pregnancy (estimated gestational age (EGA) <20 weeks); in late pregnancy (EGA >28 weeks); at three months post-partum; and at six months post-partum. Non-perinatal women will be followed-up at the equivalent time intervals. At each visit, participants will be asked to complete CMD screening tools, which will be selected on the basis of Phase 2 results. Any woman who screens positive on any screening tool will require further clinical assessment; those confirmed as having a mental disorder will be offered treatment and follow-up as appropriate. The cut-offs for each screening tool used in this phase will have been determined during the validation study. At the first visit, participants will also be asked to complete a socio-demographic questionnaire, which will include data on potential confounders as identified by reviewing the existing literature.

#### Sample size

We calculated that a minimum sample size of 1166 (583 perinatal and 583 non-perinatal) women at each site is required to demonstrate a 10% difference in the prevalence of CMD between perinatal and non-perinatal women.^35^ This assumes: best-estimate CMD prevalence of 10.7% among non-perinatal women^36^ and 20% among perinatal women;^5^ 5% level of significance; 80% power for a two-sided test; 85% retention rate over the study period; and a design coefficient of 1.29.^37^ Sample size was based on prevalence calculations because this is the primary outcome. Furthermore, this sample size is larger than what is required for risk factor analysis; our target sample size allows for multivariable regression models with up to 20 exposure variables.

#### Analysis

The presence of a mental disorder will be defined by scores above the cut-off on CMD screening tools. Point prevalence, period prevalence and incidence of CMD will be estimated in perinatal and non-perinatal groups as follows. Prevalence and incidence of CMD will be presented separately for each type of CMD (e.g. incidence of anxiety; prevalence of depression) and as a composite measure (e.g. prevalence of CMD). Univariable logistic regression analyses will be performed to calculate odds ratios to identify factors associated with CMD. Potential associated factors will be selected *a priori* on the basis of evidence from existing studies. Variables with *P*-values <0.10 will be included in a multivariable model built using forward-stepwise approach. We will use linearized or robust standard errors to adjust for clustering at the village level. If there is missing data, a complete case analysis will be conducted. Statistical advice will be sought during the analysis stage of the research as required.

### Recruitment and consent

Participants will be recruited from community settings in areas served by the two host institutions. These will include women living in low-income settings in both urban and rural areas. Recruitment at each site will be carried out by a study team of research nurses who will liaise with Accredited Social Health Activists (ASHAs), Auxiliary Nurse Midwives (ANMs) and Anganwadi workers at urban and rural sub-centres and health and nutrition (Anganwadi) centres. Non-perinatal women will be recruited through the same procedure as well as through ‘snowballing’, by asking perinatal women to identify any friends, relatives or neighbours who are of reproductive age and living in the same communities who are not currently pregnant and have not given birth within the past 12 months. Printed information sheets will be available, but as this is a low-literacy population and based on the prior research experience of the study team in similar communities, verbal explanations will form the main mode of information giving. Verbal explanations will detail the nature and purpose of the study; what the study will involve for the participant including the number and duration of visits required; the implications and constraints of the protocol; what the data in the study will be used for and any potential risks involved in taking part. It will be clearly stated that participants are free to withdraw from the study at any time, for any reason, without prejudice to future care and with no obligation to provide a reason for withdrawal. Those who choose to participate will be asked to sign the informed consent form or provide a thumbprint.

### Ethical considerations

Many of the women included in this study are likely to live in poverty and many may have low levels of literacy. In the context of Covid-19, living conditions and household incomes have become even more precarious. These vulnerabilities raise a number of ethical issues. Women we approach may not have engaged in research studies before and may not be aware of what participation entails. Care will be taken to ensure the study is explained clearly and without scientific or research jargon to enable women to autonomously decide whether to take part. Some women may feel coerced into participating from feelings of obligation, fear of discrimination or other assumed negative consequences of non-participation. Financial compensation for participation is a contested issue: if compensation is high relative to local earnings this may create an economic incentive to participate, complicating the concept of truly autonomous consent. However, for women living in poverty loss of earnings is an important consideration. Women with young children or other dependents will also be inconvenienced by participation in the study. We have therefore decided to offer all participants a payment valued at the equivalent of a daily wage at each study visit as an appreciation of their time.

As part of the study, we will identify women experiencing CMD. At both sites, referral mechanisms to mental health services are in place with capacity to deal with a potential increase in referrals. Any suspected or confirmed instances of abuse will be handled using standard operating procedures (SOP) as used in previous research in perinatal women at the NIMHANS site. SOPs are in place for two situations: domestic violence and suicidal risk. These include risk assessment and referral to appropriate agencies and services. We will only proceed with the research study when it is safe to do so and when the safety of participants is not jeopardized through travel or contact with others. We will follow local Covid-19 guidance as issued by collaborating institutions and ensure that all contact with participants occurs in line with national guidance. Ethics approval has been granted by the University of Oxford and DRPGMC Institutional Ethics Committee (IEC) and is awaited from NIMHANS IEC and India’s Health Ministry’s Screening Committee.

## Discussion

### Main findings of this study

The findings of this study will include: women’s feedback on the usability and appropriateness of selected CMD screening tools; women’s views and knowledge around CMD; assessments of the psychometric validity of CMD screening tools (including sensitivity, specificity, positive and negative predictive values and optimal cut-offs of screening tools); prevalence and incidence of anxiety disorders, depressive disorders, suicidality, PTSD and somatization disorder among perinatal and non-perinatal women; and risk factors associated with these CMD.

### What is already known on this topic

Maternal health and mental health are among the most significant contributors to persisting inequalities and morbidity and mortality globally. Perinatal CMD affects a significant proportion of women, with the highest burden carried by LMIC. Conditions such as perinatal depression and perinatal anxiety are associated with poor maternal and infant outcomes. Early detection through screening coupled with effective treatment can improve outcomes. It is essential, however, that screening tools are locally validated and culturally appropriate to ensure women with CMD can be identified.

### What this study adds

Data from this study will make a significant contribution to existing knowledge around CMD in women of reproductive age. This study will provide new knowledge on the identification of CMD across both perinatal and non-perinatal women in Karnataka and Himachal Pradesh. The screening tools we translate and validate will have potential for use in routine antenatal and postnatal care in our study settings, which scope to improve the detection of CMD in our populations. Our data will provide CMD prevalence and incidence along with associated risk factors. In particular, our study will shed light on PTSD and somatization disorder, which have been under-researched in perinatal populations in LMIC to date. Finally, by including both perinatal and non-perinatal women, the study will provide important and novel insights into the role of the perinatal state as an independent contributor to mental disorders.

### Limitations of this study

A limitation of the study is that we may not cover all income groups and educational levels among participants recruited into the study. Another limitation is that our study currently extends to six months post-partum, which precludes the investigation of infant outcomes beyond this period that may be associated with maternal CMD. However, we plan to seek additional funding to allow for longer follow-up of women and their infants in order to assess further trends in women’s mental health and associations with infant and child outcomes. A potential challenge we may have is attrition during the cohort study. Many women travel between their natal home and their partner’s home, which can lead to drop out from the study. Women recruited in early pregnancy may also experience pregnancy losses such as abortion. To address these challenges, robust systems will be developed at the time of recruitment to the cohort study to include women who have lived in the area for at least five years. We will also collect sufficient contact details to maximize follow-up, for example through telephone follow-up if necessary.

## Conclusion

Improving the understanding, detection and management of CMD among women is key to improving women’s health and promoting gender equality. The National Health Mission of India has established initiatives to include screening for mental health in maternal health settings.^8^ In order to do this effectively, there is a need for validated tools in local languages for different population groups. In addition, the study will provide incidence and risk factor data for perinatal CMD from two different parts of India and a better understanding of the specific contribution of the perinatal state to these conditions. In the longer term, there is potential to examine associations between perinatal CMD and infant outcomes and to develop culturally appropriate interventions to effectively prevent and treat CMD.

## Funding

GF is funded by a Nuffield Department of Population Health Clinical Research Fellowship. MN and MaatHRI are funded by a Medical Research Council (GCRF) Career Development Award (grant ref: MR/P022030/1).

## Supplement Funding

Oxford-India Sustainable Centre, Somerville College, University of Oxford.

## Conflict of Interest

All authors declare we have no conflicts of interest.

## Notes

**G. Fellmeth**, Clinical Research Fellow

**M.T. Kishore**, Additional Professor of Clinical Psychology

**A. Verma**, Professor of Obstetrics and Gynaecology

**G. Desai**, Professor of Psychiatry

**O. Bharti**, State Epidemiologist

**P. Kanwar**, Assistant Professor of Psychiatry

**S. Singh**, Professor of Psychiatry; Head of Department

**H. Thippeswamy**, Professor of Psychiatry

**P.S. Chandra**, Professor of Psychiatry

**J.J. Kurinczuk**, Professor of Perinatal Epidemiology; Director

**M. Nair**, Associate Professor

**F. Alderdice**, Professor of Perinatal Health and Wellbeing

## References

[1] Gelaye B, Rondon MB, Araya R et al. Epidemiology of maternal depression, risk factors, and child outcomes in low-income and middle-income countries. Lancet Psychiatry 2016;3(10):973–82. 27650773 10.1016/S2215-0366(16)30284-X PMC5155709

[2] Woody CA, Ferrari AJ, Siskind DJ et al. A systematic review and meta-regression of the prevalence and incidence of perinatal depression. J Affect Disord 2017;219:86–92. 28531848 10.1016/j.jad.2017.05.003

[3] Kishore MT, Satyanarayana V, Ananthanpillai ST et al. Life events and depressive symptoms among pregnant women in India: moderating role of resilience and social support. Int J Soc Psychiatry 2018;64(6):570–7. 30024292 10.1177/0020764018789193

[4] Kalra H, Tran TD, Romero L et al. Prevalence and determinants of antenatal common mental disorders among women in India: a systematic review and meta-analysis. Arch Womens Ment Health 2021;24(1):29–53. 32055988 10.1007/s00737-020-01024-0

[5] Upadhyay RP, Chowdhury R, Salehi A et al. Postpartum depression in India: a systematic review and meta-analysis. Bull WHO 2017;95(10):706–17. 29147043 10.2471/BLT.17.192237 PMC5689195

[6] Jyothi Kantipudi S, Kannan GK, Viswanathan S et al. Antenatal depression and generalized anxiety disorder in a tertiary Hospital in South India. Indian J Psychol Med 2020;42(6):513–8. 33354075 10.1177/0253717620928440 PMC7735237

[7] Supraja TA, Thennarasu K, Satyanarayana VA et al. Suicidality in early pregnancy among antepartum mothers in urban India. Arch Womens Ment Health 2016;19(6):1101–8. 27565804 10.1007/s00737-016-0660-2

[8] Ganjekar S, Thekkethayyil AV, Chandra PS. Perinatal mental health around the world: priorities for research and service development in India. British Journal of Psychiatry International 2020;17(1):2–5. 34287425 10.1192/bji.2019.26 PMC8277535

[9] Fellmeth G, Harrison S, Opondo C et al. Validated screening tools to identify common mental disorders in perinatal and postpartum women in India: a systematic review and meta-analysis. BMC Psychiatry [accepted] 2021;21(1):200. 10.1186/s12888-021-03190-6 PMC8056564 33879130

[10] Ali GC, Ryan G, De Silva MJ. Validated screening tools for common mental disorders in low and middle income countries: a systematic review. PLoS One 2016;11(6):e0156939. 27310297 10.1371/journal.pone.0156939 PMC4911088

[11] Shrestha SD, Pradhan R, Tran TD et al. Reliability and validity of the Edinburgh postnatal depression scale (EPDS) for detecting perinatal common mental disorders (PCMDs) among women in low-and lower-middle-income countries: a systematic review. BMC Pregnancy Childbirth 2016;16:72. 27044437 10.1186/s12884-016-0859-2 PMC4820998

[12] Stein A, Pearson RM, Goodman SH et al. Effects of perinatal mental disorders on the fetus and child. Lancet 2014;384(9956):1800–19. 25455250 10.1016/S0140-6736(14)61277-0

[13] Nanjundaswamy MH, Shiva L, Desai G et al. COVID-19-related anxiety and concerns expressed by pregnant and postpartum women-a survey among obstetricians. Arch Womens Ment Health 2020;23(6):787–90. 32839898 10.1007/s00737-020-01060-w PMC7445074

[14] Nair M, Bezbaruah B, Bora AK et al. Maternal and perinatal Health Research Collaboration, India (MaatHRI): methodology for establishing a hospital-based research platform in a low and middle income country setting [version 2; peer review: 1 approved]. F1000Research 2020;9:683. doi: 10.12688/f1000research.24923.2 (7 April 2021, date last accessed). 33500775 PMC7812614

[15] Kroenke K, Spitzer RL, Williams JB. The PHQ-9: validity of a brief depression severity measure. J Gen Intern Med 2001;16(9):606–13. 11556941 10.1046/j.1525-1497.2001.016009606.x PMC1495268

[16] Cox JL, Holden JM, Sagovsky R. Detection of postnatal depression. Development of the 10-item Edinburgh postnatal depression scale. Br J Psychiatry 1987;150(6):782–6. 3651732 10.1192/bjp.150.6.782

[17] Somerville S, Dedman K, Hagan R et al. The Perinatal Anxiety Screening Scale: development and preliminary validation. Arch Womens Ment Health 2014;17(5):443–54. 24699796 10.1007/s00737-014-0425-8

[18] World Health Organization (WHO). Management of substance abuse: process of translation and adaptation of instruments. World Health Organization, 2020. https://www.who.int/substance_abuse/research_tools/translation/en/ (7 April 2021, date last accessed).

[19] Spitzer RL, Kroenke K, Williams JB et al. A brief measure for assessing generalized anxiety disorder: the GAD-7. Arch Intern Med 2006;166(10):1092–7. 16717171 10.1001/archinte.166.10.1092

[20] Sinesi A, Maxwell M, O'Carroll R et al. Anxiety scales used in pregnancy: systematic review. BJ Psych Open 2019;5(1):e5. 10.1192/bjo.2018.75 PMC6343118 30762504

[21] Weathers FW, Litz BT, Keane TM et al. The PTSD Checklist for DSM-5 (PCL-5). 2013. www.ptsd.va.gov (7 April 2021, date last accessed).

[22] Kroenke K, Spitzer RL, Williams JB. The PHQ-15: validity of a new measure for evaluating the severity of somatic symptoms. Psychosom Med 2002;64(2):258–66. 11914441 10.1097/00006842-200203000-00008

[23] Desai G, Chaturvedi SK, Dahale A et al. On somatic symptoms measurement: the scale for assessment of somatic symptoms revisited. Indian J Psychol Med 2015;37(1):17–9. 25722506 10.4103/0253-7176.150807 PMC4341304

[24] Guest G, Namey E, McKenna K. How many focus groups are enough? Building an evidence base for nonprobability sample sizes. Field Methods 2017;29(1):3–22.

[25] Barbour RS. Doing Focus Groups. London: SAGE Publications Ltd, 2007.

[26] Braun V, Clarke V. Using thematic analysis in psychology. Qual Res Psychol 2006;3(2):77–101.

[27] Ziebland S, McPherson A. Making sense of qualitative data analysis: an introduction with illustrations from DIPEx (personal experiences of health and illness). Med Educ 2006;40(5):405–14. 16635119 10.1111/j.1365-2929.2006.02467.x

[28] NVivo. Qualitative Data Analysis Software, Version 12. QSR International Pty Ltd, 2018. https://support.qsrinternational.com/nvivo/s/article/How-do-I-cite-QSR-software-in-my-work.

[29] Sheehan DV, Lecrubier Y, Sheehan KH et al. The Mini-International Neuropsychiatric Interview (M.I.N.I.): the development and validation of a structured diagnostic psychiatric interview for DSM-IV and ICD-10. J Clin Psychiatry 1998;59(Suppl 20):22–33. 9881538

[30] Gururaj G, Varghese M, Benegal V et al. National Mental Health Survey of India, 2015-16: Summary. Bengaluru, National Institute of Mental Health and Neuro Sciences, NIMHANS Publication No. 128, 2016. http://www.indianmhs.nimhans.ac.in/Docs/Summary.pdf (7 April 2021, date last accessed).

[31] Martin CR, Redshaw M. Establishing a coherent and replicable measurement model of the Edinburgh Postnatal Depression Scale. Psychiatry Res 2018;264:182–91. 29649675 10.1016/j.psychres.2018.03.062 PMC6008486

[32] Boateng GO, Neilands TB, Frongillo EA et al. Best practices for developing and validating scales for health, social, and behavioral research: a primer. Front Public Health 2018;11(6):149. 10.3389/fpubh.2018.00149 PMC6004510 29942800

[33] Devellis RF. Scale Development: Theory And Applications, 2nd edn. Newbury Park, California: Sage Publications, 2003.

[34] StataCorp. Stata Statistical Software: Release 14. College Station, TX: StataCorp LP, 2015.

[35] Hulley SB, Cummings SR, Browner WS et al. Designing Clinical Research: An Epidemiologic Approach, 4th edn. Philadelphia, PA: Lippincott Williams & Wilkins, 2013 Appendix 6B, 75.

[36] Shidhaye R, Patel V. Association of socio-economic, gender and health factors with common mental disorders in women: a population-based study of 5703 married rural women in India. Int J Epidemiol 2010;39(6):1510–21. 21037247 10.1093/ije/dyq179 PMC2992631

[37] Mistry R, Pednekar MS, Gupta PC et al. Longitudinal study of adolescent tobacco use and tobacco control policies in India. BMC Public Health 2018;18(1):815. 29970049 10.1186/s12889-018-5727-8 PMC6029385

